# A simple high-speed random number generator with minimal post-processing using a random Raman fiber laser

**DOI:** 10.1038/s41598-021-92668-0

**Published:** 2021-06-23

**Authors:** Frédéric Monet, Jean-Sébastien Boisvert, Raman Kashyap

**Affiliations:** 1 Fabulas Laboratory, Engineering Physics Department, Polytechnique Montreal, 2900 Blvd Edouard-Montpetit, Montreal, H3T 1J4 Canada; 2 Electrical Engineering Department, Poly-Grames, Polytechnique Montreal, 2900 Blvd Edouard-Montpetit, Montreal, H3T 1J4 Canada

**Keywords:** Nonlinear optics, Fibre lasers

## Abstract

A simple novel method for random number generation is presented, based on a random Raman fiber laser. This laser is built in a half-open cavity scheme, closed on one side by a narrow-linewidth 100 mm fiber Bragg grating. The interaction between the randomly excited lasing modes of this laser, in addition to nonlinear effects such as modulation instability, allow the generation of random bits at rates of up to 540 Gbps with minimal post processing. Evaluation of the resulting bit streams’ randomness by the NIST statistical test suite highlights the importance of evaluating the physical entropy content, as bit sequences generated by this random laser pass all the statistical tests with a significance level of 0.01, despite being generated at more than twice the theoretical entropy generation speed.

## Introduction

Random number generation (RNG) has risen in interest for various applications such as Monte Carlo simulations and secure communications, where pseudo-random numbers generated by deterministic algorithms do not have the necessary non-reproducibility and non-periodicity of true physical random numbers^1^. To address this need, optical RNG based on physical phenomena has been investigated to generate random numbers at very high speeds. Chaotic semiconductor lasers are an interesting avenue, because their large bandwidth allows them to generate random numbers at extremely high bitrates^2^. Using external feedback to the cavity, RNG of hundreds of Gbps has been demonstrated using those lasers^3–5^. Furthermore, by combining multiple lasers and separating the measurements on multiple channels, RNG was demonstrated to achieve bit rates of up to 2.24 Tbps^6^.

Random fiber lasers were also investigated in recent years, for their very simple design and unique properties. While they show great promise for applications such as in speckle-free imaging^7^ and cancerous tissue diagnostics^8^, they are particularly well suited for RNG, due to their intrinsically unpredictable output. Using Brillouin random fiber lasers, RNG was demonstrated at tens of Mbps^9^. Another random fiber laser scheme using semiconductor optical amplifiers in a ring resonator achieved RNG at 1.6 Gbps^10^. However, this is still very far from the required bandwidths of high-speed secure communications and cannot compete with the high bitrates achieved by chaotic semiconductor lasers. Recent work involving ytterbium-doped random fiber lasers, relying on the Rayleigh scattering of a single mode fiber as feedback, have demonstrated bitrates of 200 Gbps^11^. This laser architecture was also used for temporal ghost imaging, demonstrating another possible application where random fiber lasers surpass the performance of conventional cavity-based fiber lasers^12^.

A common technique used to enhance RNG speeds is to post-process the acquired data. The most typically used post-processing method is to truncate the least significant bits (LSBs) from the digitized signal^3,6^. This technique requires the least amount of post-processing, can be realized in real-time, and has the additional benefit of multiplying the acquisition rate by the number of retained LSBs. Another common technique uses a delayed exclusive OR (XOR) operation on the bit streams to enhance randomness^11,13–15^. Although simple, this technique requires additional steps which can compromise real-time implementation. Even more complex post-processing algorithms have also been investigated, for example relying on successive derivatives of the measured signal, which artificially increases the number of bits digitized by an analog to digital converter (ADC). Kanter et al.^16^ showed that by computing the 15th derivative of the signal, their algorithm was able to recover 15 random bits per sample from a signal originally quantized by an 8-bit ADC. However, this complicated post-processing scheme cannot be achieved in real-time, limiting the possibility of its implementation. Furthermore, it is important to note that the use of XOR operations or numerical derivatives does not increase the physical entropy generated, which ultimately limits the attainable RNG rate. Indeed, the use of deterministic post-processing schemes can at best hide existing correlations in the dataset from statistical tests, but it cannot improve the bits’ randomness^17^.

An article recently published in *Science* by Kim et al*.*^15^ demonstrated a different RNG technique based on the interference of multiple lasing modes, both longitudinal and transverse, in a chip-scale laser diode. Due to the interaction of all the lasing modes, a total bit rate of nearly 200 Tbps was achieved by spatially multiplexing 243 channels, each generating random numbers at 820 Gbps. Each channel selected the 2 LSBs, sampling every 2.44 ps (corresponding to a 410 GSa/s sampling rate), and a self-delayed XOR operation was applied in post-processing to the acquired bits. However, it should be noted that the separation between the spatial channels (which ultimately determined the number of multiplexed channels) was selected based on statistical tests performed after the application of this XOR operation, and not on the entropy content evaluation, thus indicating the care needed to interpret the measured results.

In this work, we demonstrate a novel RNG technique that is also based on the interaction of lasing modes, but this time in a random laser. In our case, the modes are in the modeless cavity of a random Raman fiber laser, where a multitude of lasing modes will be randomly excited simultaneously. Furthermore, nonlinear effects such as modulation instability increase the inherent randomness of this laser. This laser has a very simple architecture, which relies solely on a fiber Bragg grating (FBG) and some length of optical fiber, resulting in a half-open cavity scheme. No post-processing technique other than LSB selection was used, to avoid compromising the bits’ true randomness, as well as to offer the possibility of real-time implementation. To the best of our knowledge, this is the first demonstration of RNG based on random Raman fiber lasers. It is also the highest reported bitrate for random laser based RNG, on par with current state of the art techniques such as chaotic semiconductor lasers. Furthermore, we show that, while a bit sequence generated at 1.28 Tbps can pass statistical tests such as autocorrelation and the National Institute of Standards and Technology (NIST) randomness test suite without any further deterministic post-processing steps, the theoretical entropy generated by this laser is limited to 540 Gbps. This highlights the importance of proper entropy content computation, especially for parallel RNG schemes where such tests are still heavily used to validate randomness^15,18^.

## Random Raman fiber laser emission

**Figure 1:**
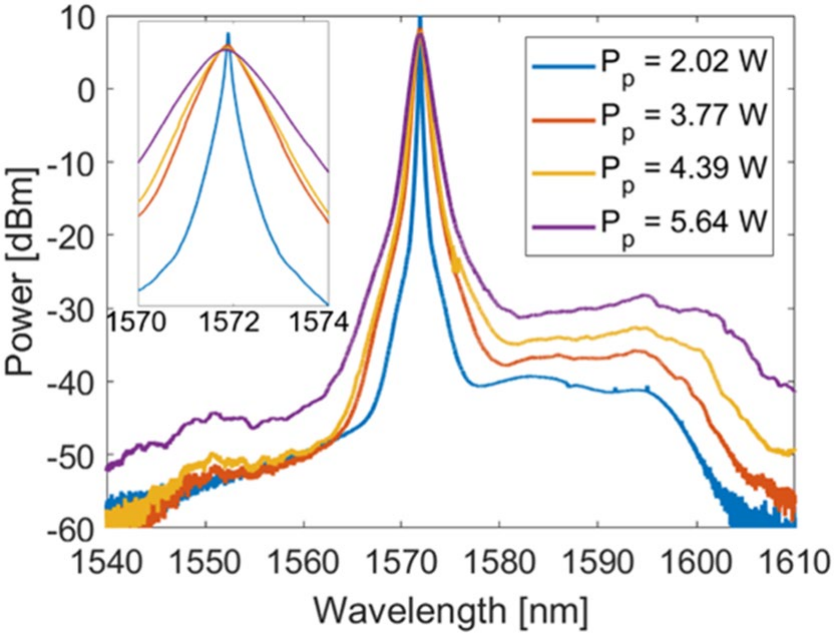
Output spectra of the random laser at various pump powers. The inset on the left shows the laser linewidth broadening.

The random Raman fiber laser is built in a very simple half-open cavity scheme comprising a highly reflective FBG and non-zero dispersion shifted optical fiber (see “Methods”). Figure 1 shows the output spectrum of the random laser at various pump powers. At lower pump powers, the laser exhibits a narrow linewidth of 320 pm at − 10 dB. However, as the pump power increases, so does the linewidth, reaching 2.66 nm at 5.64 W pump power. This laser shows 49 dB ASE suppression at low powers and 35 dB suppression at this pump power. Increasing the pump power further results in the generation of the second Raman Stokes, and as such decreases the output power at 1572 nm. Pumping near the fiber’s ZDW gives rise to nonlinear phenomena such as modulation instability. Modulation instability will break the laser output into series of ultrashort, random pulses, which, when coupled to the large number of random lasing modes in the modeless cavity structure of this laser, will further promote the random number generation process. Further details on our observation of modulation instability can be found in Supplementary Material, as well as a comparison study between the fiber used in this experiment and a standard telecommunications fiber highlighting the importance of pumping near the ZDW.

**Figure 2:**
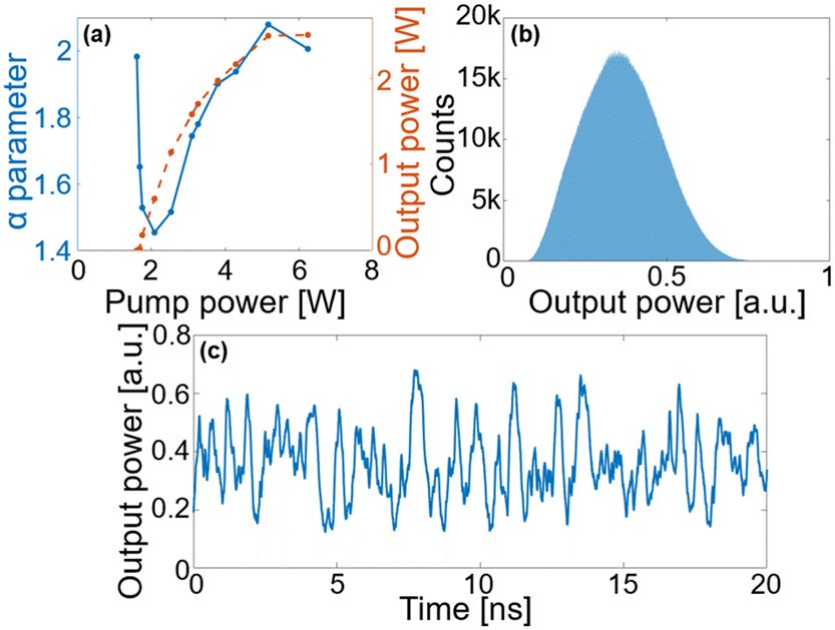
(**a**) Lévy α parameter fitted from experimental data (solid blue) and laser output power (dashed orange), as a function of input pump power. (**b**) Histogram of the output power at 5.6 W pump power, sampled over 10 million points. (**c**) Sample time sequence of the output power, at 5.6 W pump power.

To confirm that this laser is indeed operating in the random lasing regime, its output was connected to a 70 GHz photodiode, and the electrical signal was digitized by a 110 GHz, 10-bit analog-to-digital converter (ADC), real-time oscilloscope (Keysight UXR0702AP). This ADC sampled the signal at 256 GSa/s, and statistical analysis was performed on the resulting time-domain signal. Indeed, as demonstrated by Raposo et al.^19^, and later experimentally confirmed for random Raman lasers by Li et al.^20^, a random laser’s output power is characterized by a Lévy-like distribution, an asymmetrical distribution with a heavy tail towards the higher powers. The *α*-stable Lévy probability density function (PDF) is most importantly characterized by the Lévy index *α*, where *α* = 2 corresponds to a Gaussian distribution, and 0 < *α* < 2 corresponds to a Lévy-like distribution. As the pump power is increased, three different statistical regimes can be observed. Below threshold, the output follows a Gaussian distribution, with *α* = 2. However, near the threshold, the random laser’s output changes towards a Lévy-like distribution, with a sharp decrease in the *α* parameter. This change in the statistical regime of the laser has even been suggested as a universal identifier of the lasing threshold in random lasers^21^. Finally, above threshold, a gradual return towards a Gaussian regime is observed. Figure 2(a) displays the evolution of both the *α* parameter and output power as the pump power is increased. As can be observed, a sharp transition from the Gaussian regime to the Lévy regime is identified near the lasing threshold, with a gradual return towards a Gaussian regime at higher pump powers, corresponding to the expected behaviour of a random laser. The *α* parameter was estimated using the regression method proposed by Koutrouvelis^22^. Histograms displaying the distributions governing the emitted powers for increasing pump powers can be found in Supplementary Material, which highlight the transition from Gaussian to Lévy-like, and the return towards Gaussian distributions.

## Statistical randomness evaluation

To generate high quality random numbers, the distribution should be as symmetric as possible, so that the random bits are evenly distributed. In order to achieve this, a Gaussian distribution (such as the one obtained far above threshold) is preferred to the very asymmetrical Lévy-like distribution found near the threshold. As such, the pump power of 5.6 W, leading to both the highest output power and highest *α* value, was used to generate the random bit sequence. Figure 2(b) shows a histogram of the distribution of the output power in this regime. The distribution is very close to Gaussian, corresponding to a fitted *α* parameter of 2.0 ± 0.2. Figure 2(c) displays a sample time sequence of the laser output over the first 20 ns, sampled at 256 GSa/s.

**Figure 3:**
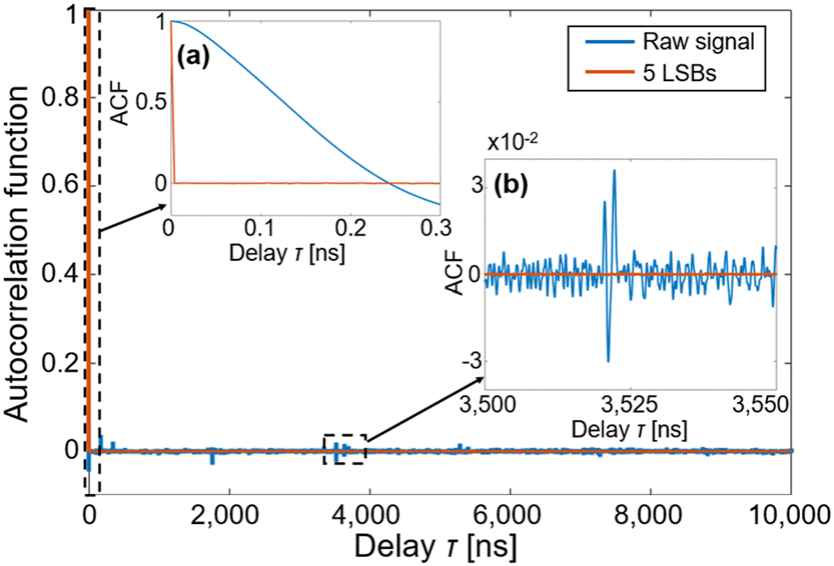
Autocorrelation of the first 10 µs for the raw signal (blue) and the 5 LSBs. Inset (**a**) shows that no correlation exists between the truncated bits after the first delay of 4 ps (since the ACF drops to 0 instantly), while inset (**b**) shows that taking the 5 LSBs removes all further existing correlation.

Using this time sequence, the 5 LSBs were extracted from the original 10 bits of the raw signal. This results in a bit rate of 1.28 Tbps (5 bits × 256 GSa/s). In order to confirm the statistical randomness of the extracted bits, the autocorrelation function (ACF) of the signal was computed. The ACF is defined as

1 $$ACF(\tau ) = \frac{{\left\langle {\left( {y(t) - \left\langle {y(t)} \right\rangle } \right)\left( {y(t + \tau ) - \left\langle {y(t)} \right\rangle } \right)} \right\rangle }}{{\left\langle {\left( {y(t) - \left\langle {y(t)} \right\rangle } \right)^{2} \left( {y(t + \tau ) - \left\langle {y(t)} \right\rangle } \right)^{2}} \right\rangle ^{{1/2}} }}$$

where *y*(*t*) is the signal as a function of time and *τ* is the autocorrelation delay. Figure 3 displays the autocorrelation of both the raw signal and the extracted 5 LSBs. As can be seen, while some correlation exists in the raw signal, taking the LSBs completely removes this correlation within the first measured delay (4 ps). After the first delay, and for the remainder of the sampled data, ACF remains less than 6 × 10^−3^, which is below the maximum value required for randomness of 1.3 × 10^−2^ experimentally observed by Takahashi et al.^13^.

**Figure 4:**
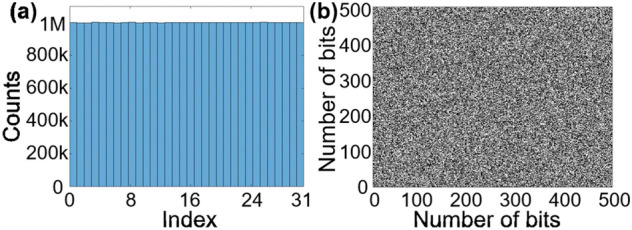
Representation of a 160 Mb bit stream. (**a**) Histogram displaying the distribution amongst the 5 LSBs. (**b**) Bit map with the first 500 × 500 random bits shown in a 2D array, where bits “0” are converted to black and bits “1” are converted to white.

Figure 4 shows the statistics of a 160 Mb bit stream resulting from this data processing. As can be seen in Fig. 4(a), the bits are evenly distributed amongst the 5 LSBs and the bit map formed by the first 250,000 bits, reshaped in a 500 × 500 array, shows no significant pattern, as displayed in Fig. 4(b).

The randomness of the data was then tested using the National Institute of Standards and Technology (NIST) Special Publication 800–22 test suite^23^, the golden standard for RNG measurements. Each test was performed over 1,000 samples of 1 Mb per sample, with a significance level 0.01. All p-values exceed 0.0001, and the proportion of successes is at least 0.980, confirming the randomness of the bit sequence. These results are summarized in Table 1, where the indicated p-values and proportions are the smallest ones in the case of multiple tests.

**Table 1:** Results of the NIST SP 800–22 for the random bit sequence generated at 1.28 Tbps.

Statistical test	p-value	Proportion
Frequency	0.419021	0.984
Block Frequency	0.664168	0.991
Cumulative Sums	0.070737	0.982
Runs	0.106877	0.989
Longest Run	0.140453	0.989
Rank	0.995777	0.990
FFT	0.008207	0.980
NonOverlapping Template	0.003224	0.980
Overlapping Template	0.637119	0.990
Universal	0.467322	0.989
Approximate Entropy	0.385543	0.992
Random Excursions	0.009604	0.983
Random Excursions Variant	0.021200	0.981
Serial	0.821937	0.991
Linear Complexity	0.347257	0.990

## Physical entropy estimation

However, statistical evaluation of the randomness cannot distinguish between true random numbers and high-quality pseudo-random numbers, such as the ones generated by algorithms. In order to determine the highest physically possible RNG rate, the entropy content must be evaluated. We base these calculations on the recommendations made by Hart et al*.* for the evaluation of photonic RNG^17^. The maximum entropy of our system is given by

2 $$h_{0} = \min \left( {\tau ^{{ - 1}} ,~2BW} \right)\left( {N_{\varepsilon } - D_{{KL}} \left( {p\left( x \right)||u\left( x \right)} \right)} \right)$$

where *τ*^−1^ is the sampling rate, *BW* is the limiting bandwidth, *N*_*ε*_ is the digitization, *p*(*x*) is the probability density function (PDF) of the entropy source, *u*(*x*) is the uniform distribution over the interval where *p*(*x*) is non-zero and *D*_*KL*_(*p*(*x*)||*u*(*x*)) is the Kullback–Leibler divergence from *u*(*x*) to *p*(*x*)^24^. Indeed, since the entropy source’s PDF is typically not uniform, a correction factor must be applied to account for this and accurately calculate the maximum entropy.

**Figure 5:**
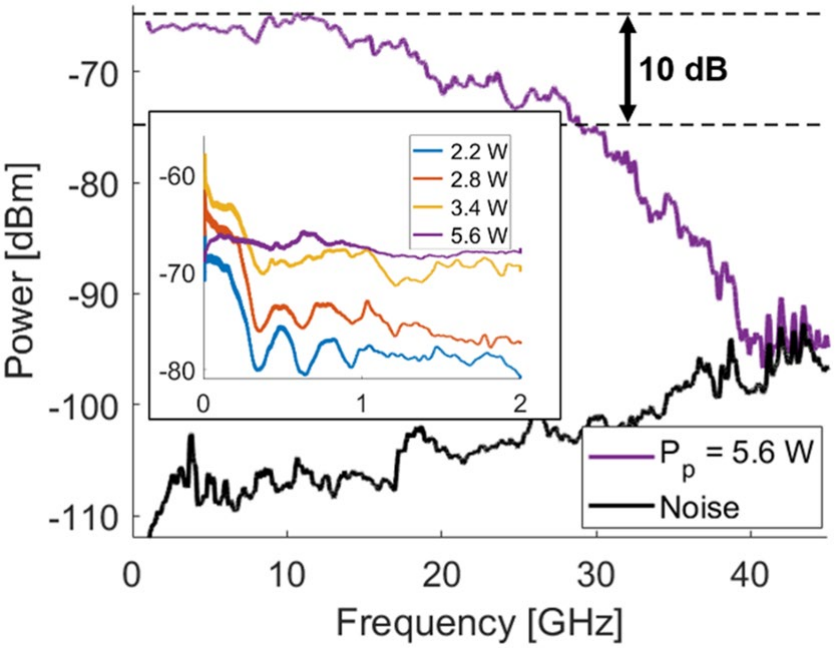
RF Spectra of the random laser over a 45 GHz span at a 5.6 W pump power. The inset shows the various peaks observed at lower pump powers, while as pump power is increased, the spectrum flatness is also improved.

The limiting bandwidth of our system can be determined by analysing the RF spectrum of the output of the laser. This was achieved by connecting the photodiode to a 50 GHz electrical spectrum analyser (Agilent PXA N9030A). Figure 5 presents the resulting spectra at different pump powers. As can be observed in the inset, at the lowest pump powers, several peaks can be observed in the RF spectrum. These peaks originate from the beating of different modes that randomly start lasing. However, as the pump power is increased, more and more modes start lasing, resulting in an increase in the number of peaks that are observed. At sufficiently high pump power, nonlinear effects such as self-phase modulation and modulation instability start appearing, leading to a broadening of those peaks. This, coupled with the increasingly large number of lasing modes in the laser, results in a very flat spectrum at a pump power of 5.6 W. This flatness extends up to 29 GHz at − 10 dB. Increasing the pump power further, as mentioned earlier, results in a decrease in output power due to the generation of the second Raman Stokes, and thus a decrease in spectrum flatness. As such, in order to maximize RNG speed, a pump power of 5.6 W should be used, to achieve maximum flatness. This corresponds with the state where the *α* value is closest to 2 (as shown in Fig. 2), where the intensity distribution is the most symmetrical. The *D*_*KL*_ was calculated from the histogram obtained in Fig. 2(b) to be 0.673 bit. Using the 29 GHz bandwidth, and with a digitization of 10 bits, this results in a physical entropy generation of 540 Gbps. This is far below the 1.28 Tbps random bit sequence that was extracted, and which passed all the NIST statistical tests. This demonstrates clearly that, as previously stated by Hart et al*.*^17^, statistical testing is insufficient for RNG validation, as the results based on it exceed by more than two times the theoretical entropy content, and may have to be revisited to establish a more rigorous test. A bit sequence generated by extracting 2 LSB (resulting in a 512 Gbps RNG rate) was also tested for validation and passed all the NIST statistical tests.

In comparison with other state of the art RNG schemes, the experimental setup we demonstrate is much simpler, being based on a single fiber Bragg grating and some length of fiber, as it does not necessitate the fabrication of complex laser diode structures^15^ or concealment of time-delay structure^6,25,26^, due to the inherent randomness of the modes in our random laser. Furthermore, in agreement with Hart et al*.*’s recommendations^17^, the only data post-processing used is least significant bits extraction to avoid tampering with the true randomness of the generated bits, and the RNG rate is based on entropy content evaluation, and not on statistical tests that, as we have demonstrated clearly, can be passed by bit sequences that exceed the generated entropy of our system. Indeed, while our bitrate of 540 Gbps may appear small in comparison to recent results such as the demonstration of nearly 200 Tbps by Kim et al*.*^15^, it should be pointed out that their massive multiplexing technique relies on many spatial channels, which are determined by statistical testing performed after deterministic post-processing. This highlights the need for a new methodology for ascertaining the physical randomness content that is generated, especially in the case of multiplexing. Using a more conservative approach, based solely on single channel RNG (i.e. one laser, one detector), the RNG bitrate we have demonstrated is either higher or comparable with the other single channel systems in literature^3,10,11,27,28^ and the bitrate of a single channel in the case of multiplexed channels^6,14,15^. With these considerations, multiplexing could potentially be implemented in the present configuration, either spectrally by using a more broadband reflector, which would allow the use of a larger portion of the Raman gain spectrum, and thus the use of multiple channels each measuring part of the spectrum, or spatially, by using for instance a multimode fiber instead of the single mode used in this experiment, which would result in a method of multiplexing equivalent to the one Kim et al*.*^15^ demonstrated, but in an optical fiber rather than in a laser diode.

In conclusion, a novel, very simple RNG scheme is demonstrated based on the interaction of randomly excited lasing modes and modulation instability in the half-open cavity scheme of a random Raman fiber laser. An entropy generation of 540 Gbps was demonstrated, and a random bit sequence generated at 512 Gbps was obtained with minimal post-processing. This new technique for producing random numbers is very easy to implement and demonstrates the potential of random fiber laser sources for RNG. The importance of calculating the physical entropy generation is also demonstrated, as statistical tests such as the NIST test suite are insufficient to confirm the randomness of a bit sequence. Indeed, we have shown here a bit sequence generated at 1.28 Tbps that passes all the statistical tests, even though the physical entropy generation of our current system is less than half this amount.

## Methods

**Figure 6:**
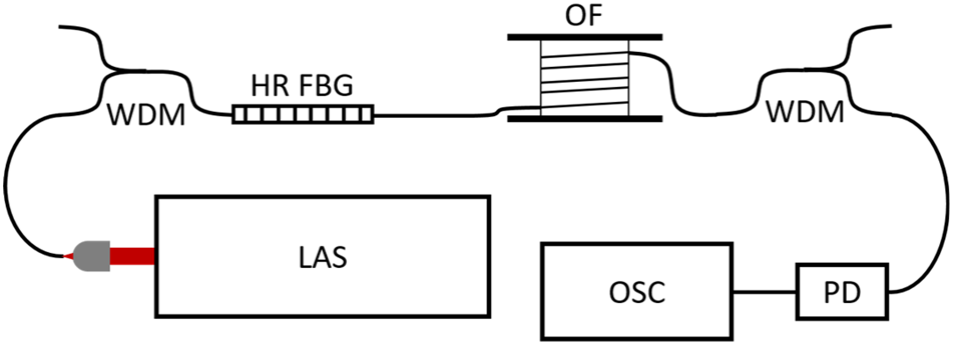
Schematic of the experimental setup, with LAS 1480 nm CW laser, WDM wavelength de-multiplexer, HR-FBG highly reflective fiber Bragg grating, OF optical fiber bundle, PD photodiode and OSC oscilloscope.

The random Raman fiber laser is built in a half-open cavity scheme which consists of a highly reflective fiber Bragg grating (HR FBG), and 6.66 km of non-zero dispersion-shifted (NZ-DS) single mode fiber (SMF-LS, Corning), as shown in Fig. 6. The laser feedback is provided on one side by the HR FBG, and on the other side by the Rayleigh scattering of the 6.66 km long optical fiber, which also provides Raman gain. The laser was pumped by a 1480 nm CW laser. This results in a high efficiency random Raman fiber laser at 1572 nm. All output fibers were cleaved at an angle of 4° to avoid parasitic reflections.

**Figure 7:**
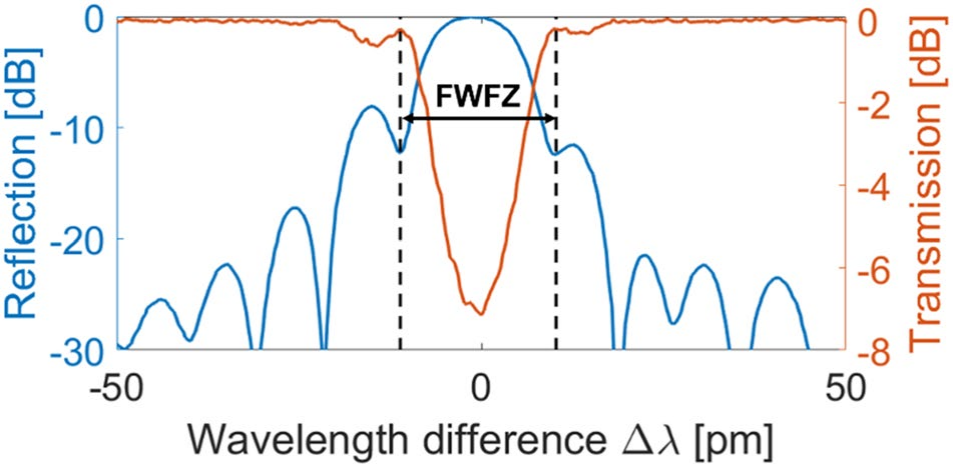
Reflexion (blue) and transmission (orange) spectrum of the FBG, showing the 21.5 pm full width at first zeroes (FWFZ) bandwidth, centered at 1571.86 nm.

The 100 mm FBG was written in standard, deuterium-loaded telecommunications fiber (SMF-28, Corning) using a Talbot interferometer writing scheme^29^, with a reflectivity of 80%. The grating was written with a wavelength of 213 nm using the 5th harmonic of a 1064 nm solid-state laser (Xiton Photonics). The FBG wavelength was set to 1571.86 nm, near the zero-dispersion wavelength (ZDW) of the NZ-DS fiber, which is around 1560 nm. A cosine apodization profile was applied on the first and last 20 mm of the grating using a phase apodization technique^30^ to mitigate the side-lobes amplitude, while maintaining a narrow bandwidth. The correction method described by Loranger et al.^31^ was used to compensate for refractive index variations along the fiber’s length. This correction allowed an excellent control of the phase of the FBG, resulting in a 21.5 pm full width at first zeroes (FWFZ) bandwidth, as can be observed in Fig. 7.

## Supplementary Information

Supplementary Information.
