# Supplementary material for: A simple high-speed random number generator with minimal post-processing using a random Raman fiber laser
Source: Sci Rep. 2021 Jun 23;11:13182. doi: 10.1038/s41598-021-92668-0 (PMC8222319; doi:10.1038/s41598-021-92668-0)
Supplement: Supplementary file 1 — Supplementary Information. [file 41598_2021_92668_MOESM1_ESM.docx]

Supplementary Material

**Random laser output power distributions at different pump powers**

As pump power increases, the probability density function (PDF) describing the output power statistics will change. Fig. S1 displays histograms of the random laser output power at different pumps, and the associated Lévy exponent *α*. At low pump powers, the random laser’s emission will be in an initial Gaussian regime, with *α* ≈ 2, as shown in Fig. S1(a). The laser’s output at that pump power is only amplified spontaneous emission, since we are below threshold. Near the threshold, the laser’s statistics will shift to a Lévy-like distribution with *α* < 2, characterised by an asymmetrical long tail towards the higher powers, seen in Fig. S1(b-c). When pump power further increases, as displayed in Fig. S1(d), the *α* exponent starts increasing again, as the PDF returns towards a Gaussian distribution. At high powers, the random laser’s emission is almost perfectly Gaussian, as seen in Fig. S1(e-f), where *α* ≈ 2 again.


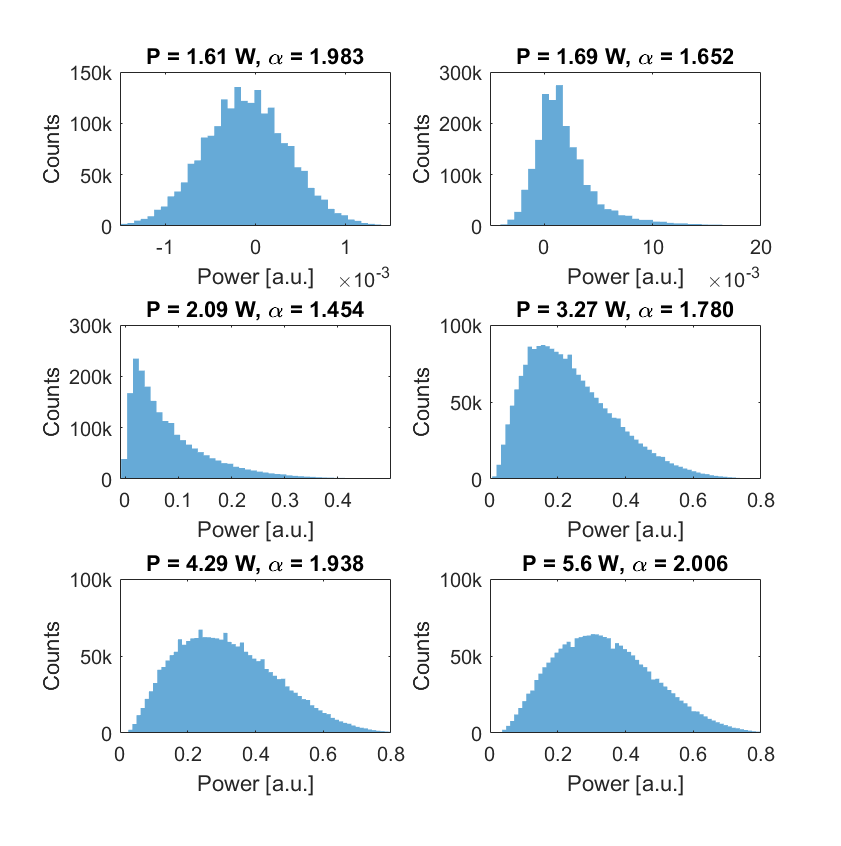


**(b)**

**(d)**

**(f)**

**(e)**

**(c)**

**(a)**

Fig. S1 Histograms of the output power at increasing pump powers. As pump power increases, the distribution is shown to shift from Gaussian (a), to Lévy-like distributions (b-d), with a return to Gaussian (e-f).

**Spectrum of NZ-DS and TF optical fibers**

In order to demonstrate the importance of pumping near the fiber’s zero-dispersion wavelength (ZDW), the non-zero dispersion shifted (NZ-DS) optical fiber (SMF-LS, Corning) used for the cavity was replaced by 6 km of standard telecommunications fiber (TF) (SMF-28, Corning). The same fiber Bragg grating (FBG) was used in both experiments. While the TF’s ZDW is around 1300 nm, the NZ-DS fiber’s is near 1560 nm, which is much closer to the 1572 nm lasing wavelength, dictated by the FBG used in the experiment. Fig. S2 compares the spectrum of both lasers. As can be easily seen, in order to achieve the same linewidth broadening, a much higher pump power is necessary for the TF than for the NZ-DS fiber. This is not surprising, as the MI frequency is given by

$\Omega_{MI}=\pm\sqrt{\frac{2\gamma P_{0}}{\left| \beta_{2} \right|}}$, **(S1)**

where *γ* is the fiber’s nonlinearity, *P_0_* is the lasing power and *β_2_* is the fiber’s group velocity dispersion parameter. Since in the case of the NZ-DS fiber, the lasing wavelength is near the ZDW, the *β_2_* parameter is much smaller than for the TF. As such, the same MI frequency can be obtained at lower pump powers.


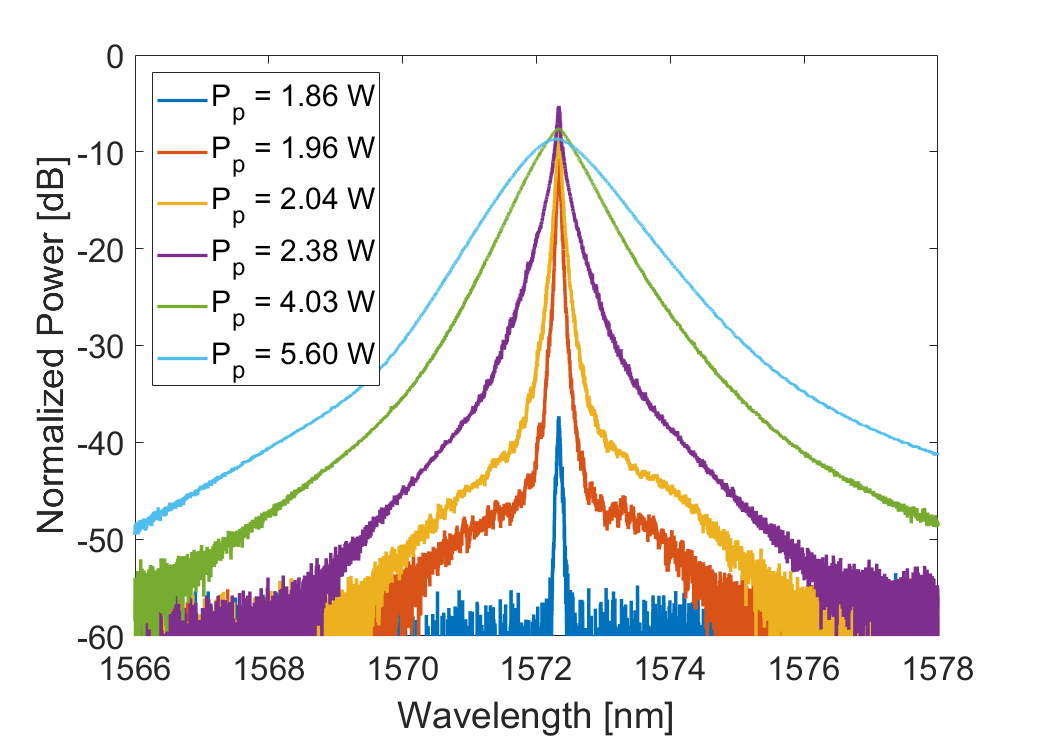

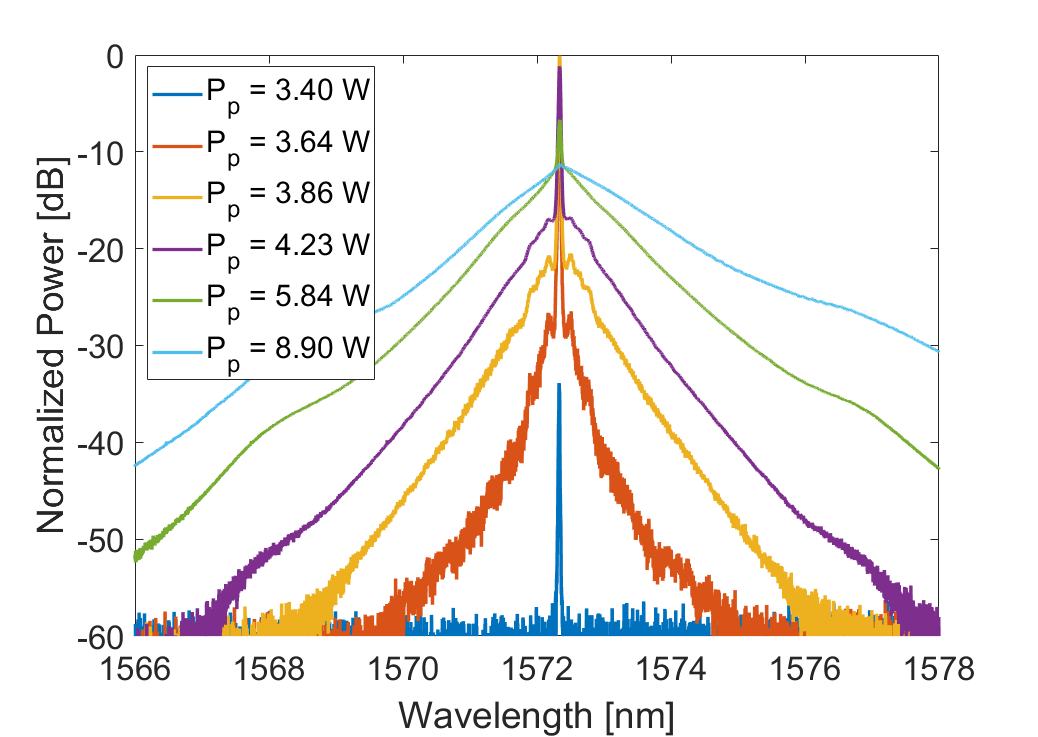


**(b)**

**(a)**

Fig. S2: Comparison of the spectrum when using (a) NZ-DS optical fiber and (b) standard telecommunication fiber.

Using Eq. (S1), and the specifications of telecommunications fiber SMF28 (*β_2_* = -25.3 ps^2^/km, *γ* = 0.78 W/km), and an output power *P_0_* of 1 W, the theoretical modulation instability frequency is 2.52×10^11^ rad/s, which corresponds to a wavelength detuning of 0.33 nm at 1572 nm. This is in line with the observed side-lobes of Fig. S2(b). For the SMF-LS fiber (*β_2_* = -1.58 ps^2^/km), the side-lobes are expected to appear near ±1.3 nm. Due to the important broadening of the laser, those side-lobes are challenging to observe. However, at the lowest pump powers, a symmetrical broadening can be observed at the calculated MI wavelengths, suggesting the presence of MI.
